# Being fully digital: perspective of a Dutch academic pathology laboratory

**DOI:** 10.1111/his.13953

**Published:** 2019-09-12

**Authors:** Nikolas Stathonikos, Tri Q Nguyen, Clothaire P Spoto, Marina A M Verdaasdonk, Paul J van Diest

**Affiliations:** ^1^ Department of Pathology University Medical Centre Utrecht Utrecht The Netherlands

**Keywords:** diagnostics, digital pathology, digital primary diagnostics, implementation, laboratory management

## Abstract

The introduction of fast and robust whole slide scanners has facilitated the implementation of ‘digital pathology’ with various uses, the final challenge being full digital diagnostics. In this article, we describe the implementation process of a fully digital workflow for primary diagnostics in 2015 at the University Medical Centre in Utrecht, The Netherlands, as one of the first laboratories going fully digital with a future‐proof complete digital archive. Furthermore, we evaluated the experience of the first 2 years of working with the system by pathologists and residents. The system was successfully implemented in 6 months, including a European tender procedure. Most pathologists and residents had high confidence in working fully digitally, the expertise areas lagging behind being paediatrics, haematopathology, and neuropathology. Reported limitations concerned recognition of microorganisms and mitoses. Neither the age of respondents nor the number of years of pathology experience was correlated with the confidence level regarding digital diagnostics. The ergonomics of digital diagnostics were better than those of traditional microscopy. In this article, we describe our experiences in implementing our fully digital primary diagnostics workflow, describing in depth the implementation steps undertaken, the interlocking components that are required for a fully functional digital pathology system (laboratory management, hospital information systems, data storage, and whole slide scanners), and the changes required in workflow and slide production.

## Introduction

The introduction of fast and affordable whole slide scanners has facilitated the implementation of ‘digital pathology’: viewing digital whole slide images (WSIs) on computer screens instead of using the traditional microscope. The utility of digital pathology includes archiving,^1^ research,^2^ teaching,^3^, ^4^ facilitating multidisciplinary meetings,^1^ remote diagnosis, e.g. for frozen sections,^5^ remote consultation,^6^ and primary diagnostics.^7^, ^8^, ^9^, ^10^ It is therefore not a surprise that the overall adoption of digital pathology has increased in the past few years, given the benefits offered by the available technology. Laboratories have been able to share and consult on cases with colleagues, archive interesting cases, provide educational modules, and facilitate research projects. Digitisation of glass slides has enabled laboratories to avoid shipping glass slides around, which permits laboratories to hold onto the physical glass slides, and instead send out digital copies. Large review research projects can be performed more quickly by having all cases digitised and hosted on a central location, allowing access for pathologists to review cases independently of their location.^11^ Pathologists can review cases from the convenience of their home or while being remote at, for example, meetings.

Over the past few years, digital pathology has become more widely adopted in pathology laboratories around the world. Especially in The Netherlands, many pathology laboratories have at least some affinity with digital pathology, either by owning a digital slide scanner, participating in slide panels hosted on digital pathology systems, or attending/providing educational lectures based on digital slides. Of the 45 pathology laboratories in The Netherlands, 16 own a digital slide scanner, including the eight academic pathology laboratories, and many more have serious plans to buy hardware. However, the adoption of digital pathology for primary diagnostics has remained relatively low, despite widespread validation of digital pathology for primary diagnostics.^12^, ^13^

The University Medical Centre (UMC) Utrecht committed 10 years ago to building up a digital pathology infrastructure based on three Aperio scanners (Leica Biosystems, Wetzlar, Germany), a tape storage system, and custom in‐house‐developed image integration software, amassing a complete digital archive of scanned histology slides over the last 10 years.^1^ As of the end of 2015, the total amount of digital slides scanned and archived in the UMC Utrecht digital slide archive (including slides scanned for research) was in the order of 500 terabytes, with >1 200 000 million scanned slides being archived. The infrastructure was set up to accommodate all useages except for primary diagnostics: archiving,^1^ to facilitate digital multidisciplinary meetings, teaching,^3^, ^4^ research,^2^ and digital quality control. We also set up a server using the Aurora system for remote consultation and slide panels.^14^

Although this was a breakthrough and has allowed us to adapt to working digitally for many years, most of the hardware was to be written off in 2015, which urged us to evaluate limitations of the infrastructure at the time and our future ambitions at the beginning of 2015. The scanners were not able to scan multiple focus planes, so we did not scan any cytology slides. There was no systematic quality control of digital slides. The scanners were too slow at scanning slides for primary diagnostics without significant delays during peak hours, and we had no professional workflow software solution to manage case assignment for pathologists and residents and performing primary digital diagnostics. Therefore, we decided that it was time for the next step. As we had extensively and successfully evaluated WSIs for primary diagnostics, we formulated our new ambition: a professional highly efficient workflow system for primary digital diagnostics without negative effects on throughput time, ready for implementation of image analysis/artificial intelligence, while maintaining our principle of a complete digital archive. Here, we review the process that we went through to become fully digital, a learning experience that other laboratories may profit from.

### The Tender Process

The new system needed to be a complete overhaul of the old digital pathology system, which meant a significant upfront investment to cover new scanners, IT hardware (servers, storage, computers, and screens), an image management system, and the costs of the implementation project. The amount of capital investment needed and the fact that our institution is publicly funded warranted a European tender process to select the vendor. The file storage part for the archive was separately funded and was kept out of the tender, as was the hardware for hosting the resulting digital pathology system, because that would be part of the regular IT infrastructure.

We opted not to follow the traditional tender process, i.e. compiling pages of technical characteristics that the solution had to adhere to and ending up selecting the lowest bid, but instead followed a ‘best‐value procurement’^15^ procedure, whereby we defined a limited set of mandatory requirements that the solution had to adhere to (Table 1), and challenged the vendors to submit a proposal that especially highlighted the added value of their solution. We set a maximum ‘total cost of ownership’ price for a 5‐year period to ensure that the costs would fit within our investment and consumables budgets. The bids had to comprise four parts (Table 2), which would be independently assessed according to upfront and transparently set criteria by a committee composed of a senior purchaser from the UMC Utrecht, an external digital pathology consultant, the IT manager, and the head of department of UMC Utrecht Pathology. Above‐average scores on the four parts would translate into a virtual reduction of the quotation of each vendor, whereby, in the end, the vendor with the lowest virtual price would win the tender.

**Table 1:** Mandatory requirements in a ‘best‐value procurement’ tender procedure for implementation of a fully digital workflow at the University Medical Centre (UMC) Utrecht, The Netherlands, in 2015

	Mandatory requirement
1.	The scanner should be able to scan multiple layers (Z‐scanning; 3D scanning) in order to be able to scan cytological slides in the future. This feature should be available for the UMC Utrecht within 2 years. Describe in the performance motivation the current status of this feature and the planning for the next 2 years
2.	The system provides an optimum archiving strategy. It should be possible to archive images for the long term with an automatic method, e.g. by increasing compression or saving in lower resolution. Describe in the performance motivation how this system will be realised
3.	The system can save images and/or export to the DICOM format (as specified by supplement 145). The support for DICOM should be available for the UMC Utrecht within 1 year. Describe in the performance motivation the current status of the feature and the planning for the next 2 years
4.	The total scanner capacity should be sufficient to scan the daily slide production of ~800 slides and a peak production of 120 slides per hour with a theoretical average tissue size of 15 × 15 mm. In the performance motivation, provide an explanation of the scanning speed in relation to tissue size. In the realisation phase, there will be a verification of the scanning speed by use of a representative dataset of glass slides provided by the UMC Utrecht
5.	The solution provides an uptime guarantee of 98% during ‘normal work hours’ on weekdays of the department. (Monday to Friday from 7.30 to 17.00). In the performance motivation, provide the uptime guarantee

**Table 2:** Overview of the four parts that vendor bids had to comprise to be independently assessed in a ‘best‐value procurement’ procedure for implementation of a fully digital workflow at the University Medical Centre Utrecht, The Netherlands, in 2015

Risk dossier	An overview of the risks identified by the vendor during the life of the project, along with a mitigation strategy
Opportunity dossier	An overview of all of the opportunities that the vendor can identify during the life of the project. This includes all of the extra features, products and services that might be relevant to the success of the project but are not part of the tender itself
Offer dossier	The offer of the proposed solution; all products, services and features included in the tender
Performance motivation dossier	Explanation of all of the features, products and services that the vendor will provide, as well as technical details

The tender was explicitly defined as a partnership for 5 years, so one of the requirements was for the vendors to treat this as an intended warm partnership in which the delivered system would be further developed and improved, and our site could be/would be used a test site for further modification and optimisation of the system, which was likely to grow far beyond the original specifications. Among the requirements was that the system delivered would include scanners with enough capacity to scan the daily production and cover slides produced during peak hours: at least 800 slides per day and 120 slides per hour. In this way, the throughput of the system would not act as a bottleneck for the diagnostic workflow.

Five offers were received and fully evaluated in the tender. Eventually, a consortium composed of Sectra AB (Linköping, Sweden) and Visiopharm (Hoersholm, Denmark) (as the reseller of Hamamatsu Photonics K.K. in The Netherlands) came out as the winner, proposing a system based on Hamamatsu (Hamamatsu City, Japan) scanners, Sectra PACS as the image management and workflow system, and Visiopharm image analysis software. Project management would be the responsibility of Sectra, which is experienced in similar processes in radiology. This solution was further evaluated during the acceptance phase, after which the contract would be final when all acceptance criteria (Table 3) were met.

**Table 3:** Acceptance criteria according to which the implemented digital diagnostics solution at the University Medical Centre Utrecht was evaluated

	Acceptance criteria
Scanners	
1.	Scanners can scan at least 800 slides per day
2.	At least 120 slides per hour (peak production)
3.	One scanner for 2 × 3 slides and fluorescence
4.	Z‐stack scanning
Laboratory workflow	
1.	Slide production turnaround times are similar to those with the current workflow (delivery of physical slides versus digital)
2.	It takes technicians no more than 2 min per batch to complete all slide handling (loading the slides onto the rack, and starting the scan process)
3.	The proportion of rejected scans should not be >2%
4.	Can scan slides in priority order when needed

### Slide Scanners

The proposed solution offered by the vendors was three Hamamatsu XR scanners and one Hamamatsu RS scanner with a fluorescence unit. The Hamamatsu XR scanner has a loading capacity of 320 slides, with an effective scanning time of 60 s per slide for a 15 × 15‐mm tissue slide, and can also perform Z‐stack scanning. The Hamamatsu RS scanner has a capacity of six slides or two 2 × 3 slides. The fluorescence unit houses a mercury lamp with a wide spectrum of light emission. It can accommodate up to six excitation filters, six emission filters, and two cube changers, which allows for a large combination of fluorescence imaging.

The scanners were thoroughly tested during the criteria acceptance phase to determine whether they would perform as described above in the real world. We selected a set of representative slides from the daily workload for testing of the scanners. The size of the tissue was, on average, slightly greater than what was requested during the tender: 18.5 × 18.5 mm. Taking into account scanning and handling time, the XR scanners were able to scan 43 slides per scanner in 1 h, taking into account slides for which scanning might have failed. During this testing scenario, the scanning performance of the scanners was deemed to be adequate.

While obtaining experience with the scanners and viewer, we noticed that the scanners needed different scan profiles to successfully scan all slides. Therefore, in total, 10 different scanning profiles were developed, for haematoxylin and eosin (H&E)‐stained slides, immunohistochemically (IHC) stained slides, special stains, fatty tissue, fluorescence, etc. We implemented a system in which, on the basis of the barcode information read from the slide, the scanner automatically chooses the appropriate scanning profile. The main differences between profiles relate to tissue detection thresholds (contrast), division of scan areas, and number of focus points per scan area.

**Figure 1:**
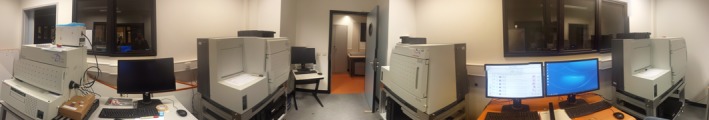
The University Medical Centre Utrecht scanner room.

The scanners were placed in a separate room, and all four appeared to produce substantial amounts of heat, noise, and ozone. This necessitated the use of extra climate control and suction, after which the working circumstances were excellent (Figure 1). This, and a lack of space in the histology laboratory, precluded the placing of scanners next to the staining/coverslipping machine, which would have had logistic advantages.

**Figure 2:**
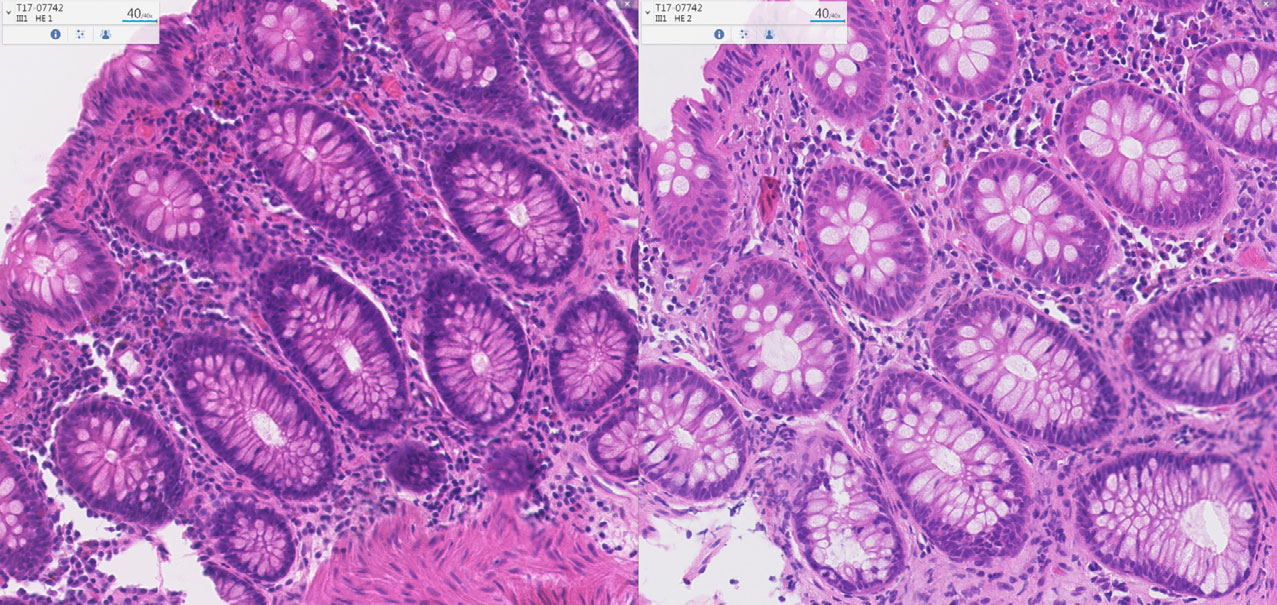
Differences in the quality of whole slide images between 4 μm and 3 μm in thickness. Left image: slide, 4‐μm section. Right image: 3‐μm section. Note the increase in contrast and how much easier it is to distinguish between individual nuclei.

Shortly after the system had gone live, problems with image quality were perceived, precluding a digital diagnosis in some areas. This appeared to be related to the limited numerical aperture of the scanner lenses, leading to fairly thick optical slices blurring nuclear detail. This hindered diagnosis, especially for gastrointestinal biopsies, in which *Helicobacter pylori* and intraepithelial lymphocytes were difficult to see, and dysplasia could be overdiagnosed. Furthermore, counting of mitoses was more difficult, and this was particularly problematic for brain tumour diagnosis and breast cancer. A workaround was found by making the gamma adjustable in the PACS system and switching from 4‐μm‐thick to 3‐μm‐thick sections (Figure 2).

### Infrastructure

The PACS system was designed for high availability and minimum downtime, which requires a high‐availability IT infrastructure and a fully redundant environment to sustain operations. This is evident in radiology departments, where the PACS system is widely used 24/7, and, especially in departments such as emergency care, the downtime afforded is zero. Pathology departments have no such high standards regarding the availability of IT systems, as working hours are usually 10/5, and, during working hours, downtime can be afforded because of the use of microscopes and emergency procedures (hand‐staining). When a system goes offline in pathology, emergency procedures are triggered, with usually minimal impact, whereas, in radiology, downtime usually means that no diagnostic tasks at all can be performed. However, when a PACS system is used as the primary workflow in a diagnostic pathology setting, any downtime or bug or feature not working correctly is perceived more severely by users than are known issues and bugs in known systems. It is therefore important for end‐user experience to introduce systems as seamlessly as possible, which we noticed when, in one instance, the system became slow because of a network cable not being well seated. This entails minimising the perceived downtime with fully redundant hardware and high‐availability systems. The tender offer guaranteed an uptime of 99.9%. This could be realised by the active monitoring and remote intervention services offered by Sectra from Linköping, providing surveillance for all Sectra pathology and radiology clients.

The PACS system is hosted on a VMware cluster running on three physical servers as an active–passive configuration. The cluster hosts two nodes so, in the case of system failure, the other node comes online. No adjustments to the standard 1‐gigabit hospital network connections and switches appeared to be necessary; streaming technology for image retrieval was excellent even through Wi‐Fi.

### Pathologists' Workstations

**Figure 3:**
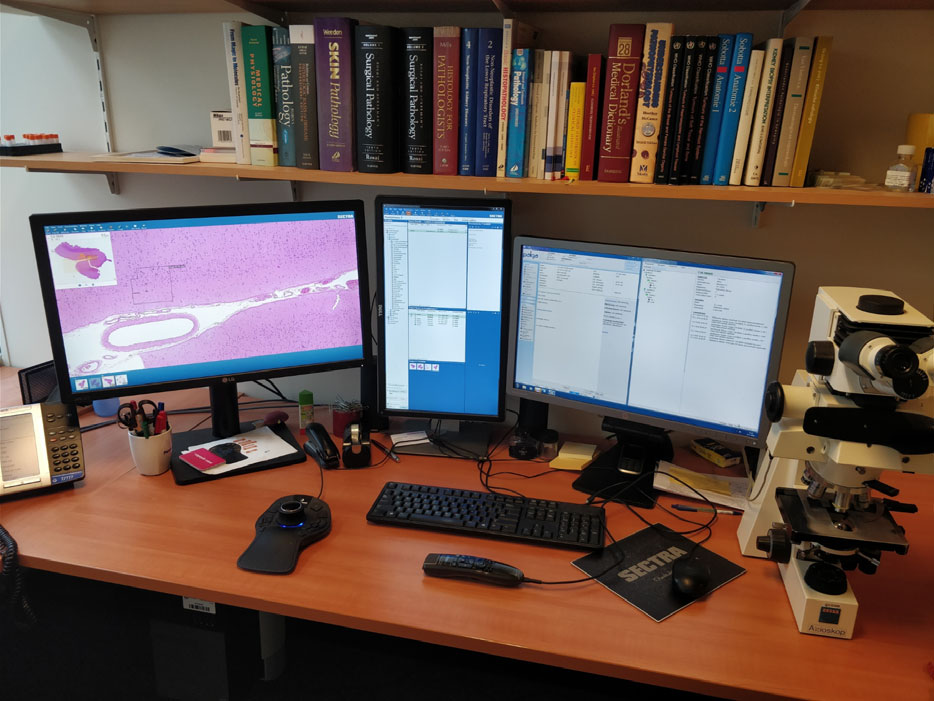
The University Medical Centre Utrecht pathology ‘cockpit’ for digital diagnostics.

Every diagnostic workstation was fitted with three screens, one of which was a 27‐inch 8.3‐megapixel display, with an IPS panel being used as the main image viewer, and two extra screens being used to display the pathology reporting system as well as the PACS information screen (Figure 3). The resolution of the main viewer display surpasses that of the diagnostic microscope at an equivalent magnification^16^ as well as an equivalent field of view (FOV). This makes the digital display superior than the microscope in terms of FOV.

Every workstation was fitted with a 3D Spacemouse Pro (3DConnexion, Munich, Germany), which is used to navigate the digital slides in the image viewer, next to the standard mouse. The seamless integration of the 3D Spacemouse with the PACS viewer offers flexibility in working with the system, which actually enhances user experience rather than making it more complicated. Usually, input devices work on the window that is active at that moment, so, when a user clicks on another window, e.g. the patient information system, the input device no longer responds. However, the 3D Spacemouse is always tied to the image window, so it is independent of which window is active. This permits the pathologist to navigate the digital slides even if the pathologist is using other applications at the time. The several button shortcuts give access to advanced navigation features to the user (picture), rendering the user's interaction more efficient and pleasant.

Several pathologists had their room layout changed to accommodate double‐scoping with residents, as this would be performed primarily digitally. In several rooms, we added extra screens to project the wholeslide viewer. In our diagnostics room, we added extra screens to allow projection of digital slides during sessions at the multiheaded microscope.

Pathologists are prone to several types of injury related to their working conditions, e.g. neck injuries due to prolonged microscope use, so we wanted digital workstations to not add to that list of occupational injuries; instead, we wanted to use them as an opportunity to reduce it. A great deal of attention was given to the ergonomic setting of the workstation, including working with residents. We also wanted to use this opportunity to improve the daily ergonomics of every pathologist by offering a better experience than the traditional one. By placing the screens at an optimal distance in a custom configuration per pathologist and placing a 3D mouse, we seem to have reduced the number of complaints related to neck/shoulder pains and carpal tunnel syndrome.

### Storage

Storage was set up as a two‐tier system, with the first tier being set up on an EMC VNX5200 low‐latency and high‐bandwidth storage system, which provides local storage for the VM cluster (OS, databases), as well as the short‐term storage for digital slides. The second tier storage is an EMC Isilon (Dell, Round Rock, TX, USA) hospital‐wide system, which is highly scalable (up to petabytes) and highly redundant, and in which images are stored after being archived. All scanning for diagnostic purposes is performed at ×40 to provide optimal‐quality images. It is our view that, for maximum clinical efficiency, all images need to be kept. However, in order to keep the storage affordable, we intended to implement a storage reduction strategy by employing an archiving system that would strip the ×40 layer from the images at the time of second‐tier storing (after completion of diagnosis). This data reduction technique has not yet been implemented, as its clinical impact has not yet been evaluated. We are still performing technical evaluation of it. Currently, the majority of the scanned images are saved in the native Hamamatsu NDPi format, so our archive consists mainly of a proprietary format. Ideally, we would like to have everything saved as DICOM objects, but, owing to operational issues, we have yet to implement this. This requires that every scanned file be converted to DICOM format after it is scanned, adding an extra step to the process, whereas, ideally, we would like to have everything scanned directly to DICOM format. This is a feature that the current generation of scanners do not yet support.

However, this limitation does not apply to our already scanned archive of Aperio images (Leica Biosystems). As part of the contract, the 8‐year archive of images is being converted to DICOM format to be available through the PACS system. Our complete archive will be stored in DICOM format, making it interoperable with future systems (such as a hospital‐wide vendor‐neutral archive) and migrations.

### Image Management System

As soon as the slides are scanned, they are copied to an import folder that is linked to the PACS system. The import service reads the unique slide ID on the 2D datamatrix barcode of the label of each slide, and, on the basis of the ID, it retrieves all slide information from the laboratory information management system (LIMS), and imports the slide to the relevant case. All image data are then imported to the PACS system.

### Integration with Laboratory Management, PALGA and Hospital Information Systems

**Figure 4:**
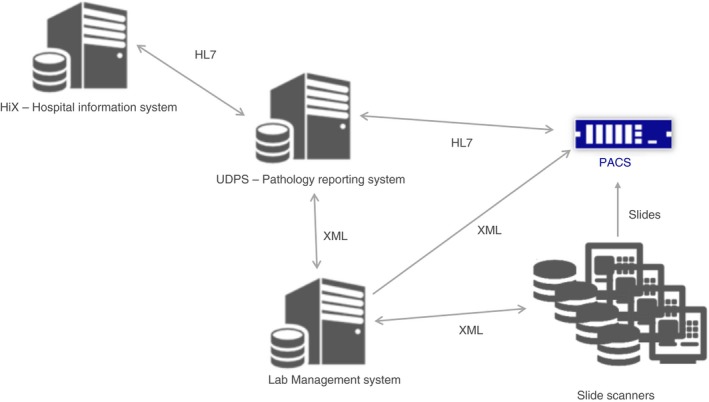
Overview of the different connections between the components of the University Medical Centre Utrecht digital pathology diagnostics solution.

Several new connections needed to be made to run the PACS system as the primary workflow system (Figure 4). The most important were those between the Universal Decentral PALGA system (U‐DPS, the local pathology reporting system) and the PACS system, between the PACS system and the LIMS (Finalist, Groningen, The Netherlands), between the LIMS and the slide scanners, and between the PACS system and the slide scanners. These connections were largely based on Integrating the Healthcare Enterprise standards and, more specifically, on standard Health Level Seven protocols.

### Viewer

**Figure 5:**
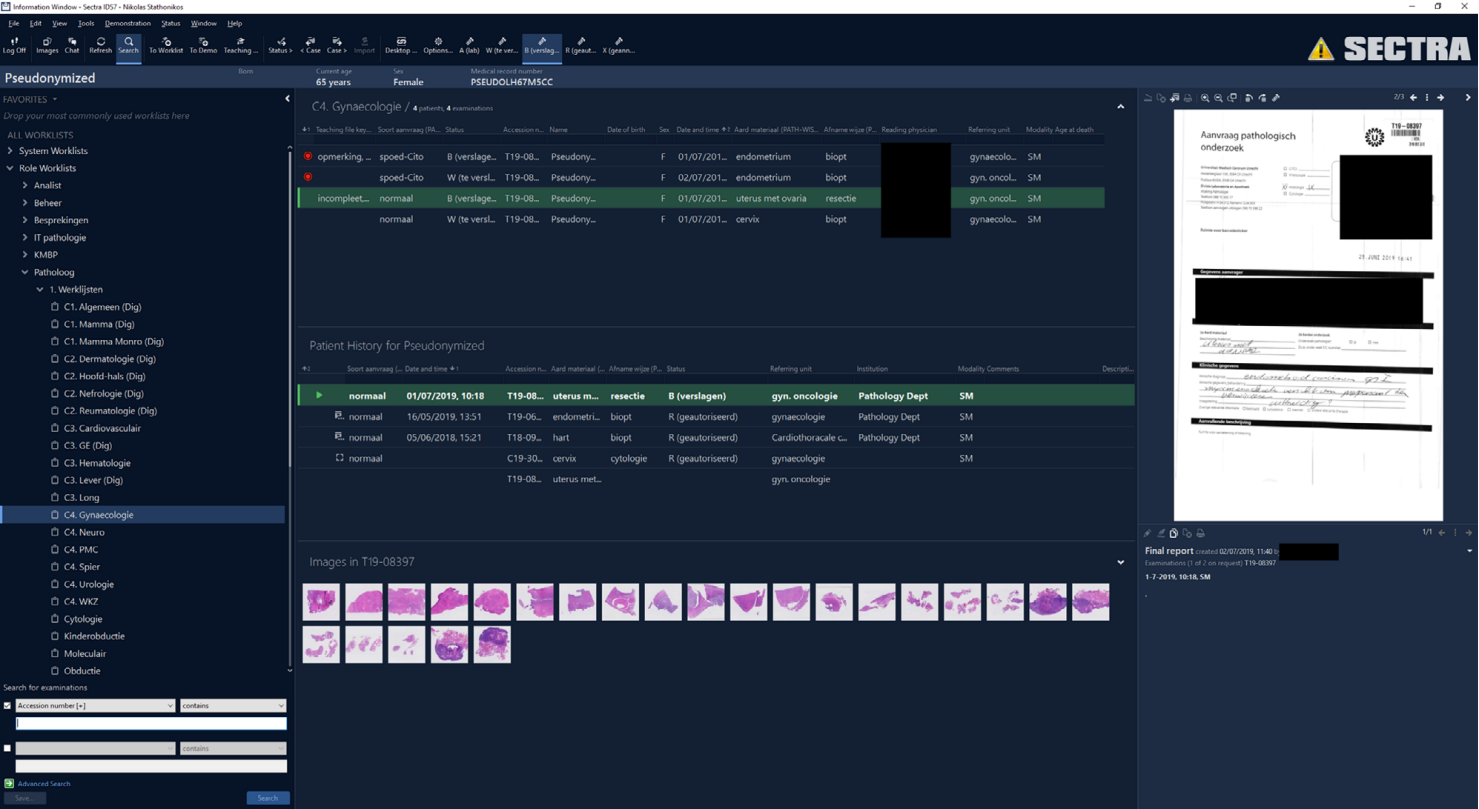
Overview of the various panels in the PACS information screen.

The viewer interface of the PACS system consists of two screens. One of these is the information screen, consisting of multiple panels (Figure 5). The leftmost customisable panel allows the user to browse all folders, in which cases can be organised by date, organ type, by urgency, or in any other ways that the user desires. When one of these folders is opened, all cases assigned to the folder are shown in the upper‐middle panel. Here, identifying information such as case ID, patient name, date of birth, and gender, is mentioned. The treating physician and the pathologist assigned to the case are also listed here. When one of the cases is selected, the patient history appears in the bottom‐middle panel, showing all previous resections and biopsies, and associated images such as macroscopy pictures and X‐ray images. This allows for fast switching between multiple submissions of one particular patient. In the rightmost panel, scanned documents (request forms and drawings) belonging to the selected case can be viewed.

**Figure 6:**
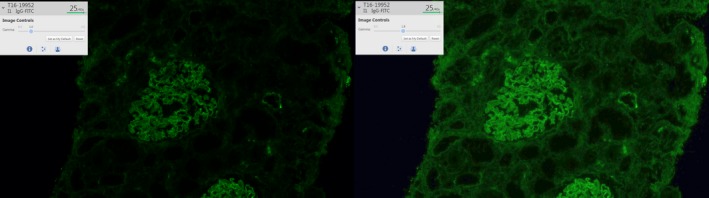
Influence of gamma correction on the quality of fluorescent images. Left: image with gamma set at 1.0. Right: image with gamma set at 1.8. Note how many more structures are visible after the gamma value is changed.

**Figure 7:**
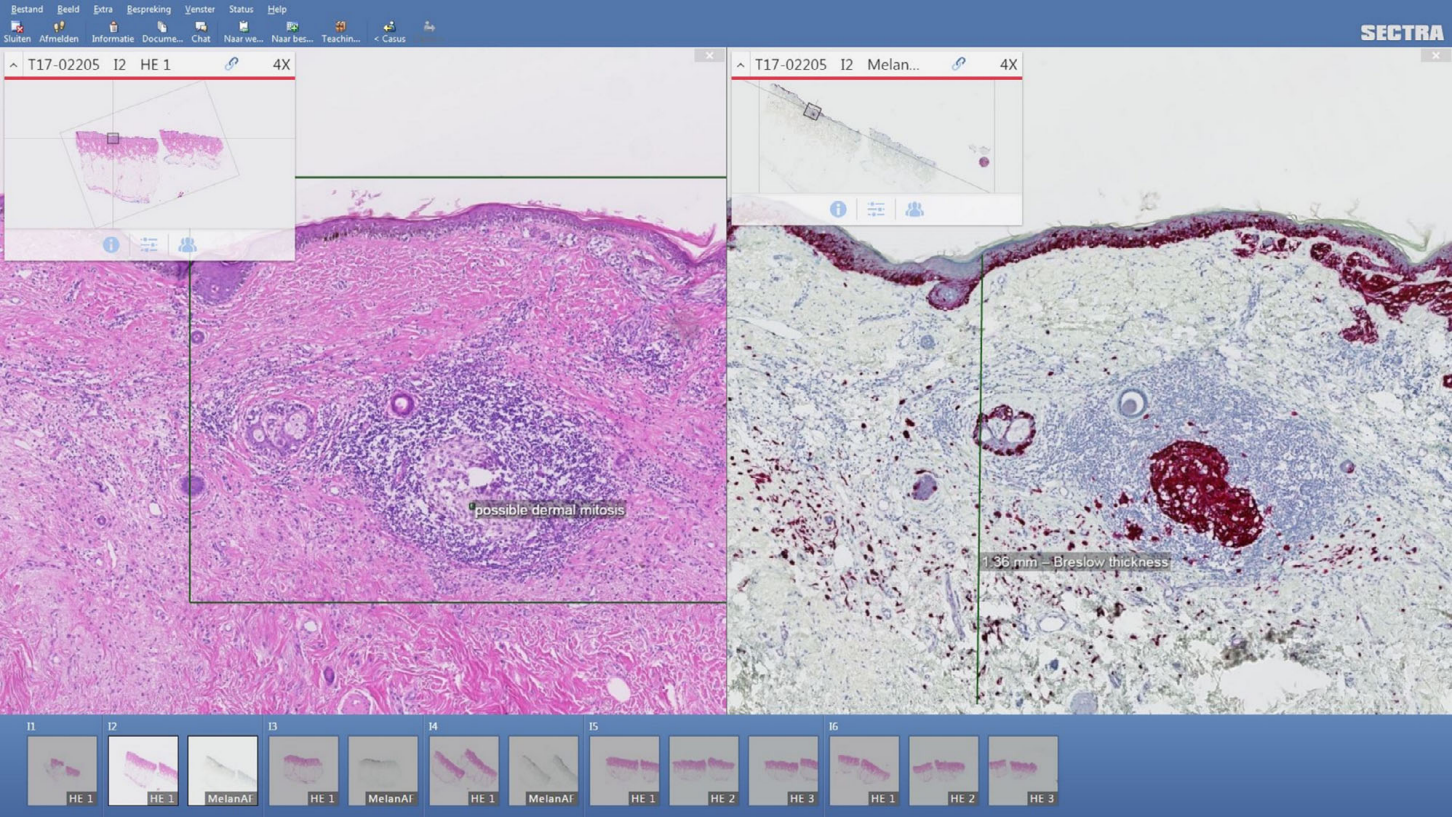
Viewing different stains side‐by‐side in the PACS viewer. Multiple images can be synced in the same view pane, and changes in magnification applied to one image are automatically apply to all other images.

**Figure 8:**
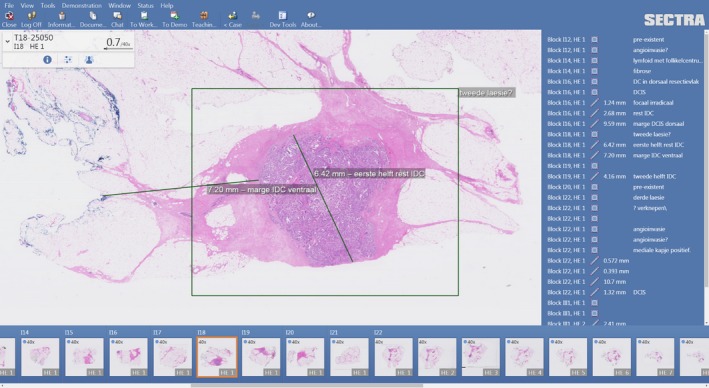
Example of annotations in the PACS viewer.

Upon double clicking of one case, the second (image) screen opens. All slides associated with the case—including H&E‐stained slides, immunohistochemical slides, and immunofluorescence slides—are shown as thumbnails at the bottom. When a slide is selected, the navigation pane providing an overview of the whole slide is shown at the top left, including information concerning current viewing magnification, the slide tag, and a menu for adjusting optical options to improve image quality. In Figure 6, gamma correction has been applied to provide a crisper immunofluorescent image. Viewer magnification ranges from ×3 to ×800 (digital zoom). The image can be rotated 360°, and multiple slides (up to four) can be viewed simultaneously, providing a useful tool with which to compare different stains in the same area (Figure 7). An algorithm in the PACS software enabling ‘side‐by‐side viewing’ ensures that the multiple images are locked in the same view pane and that changes in magnification applied to one image are automatically applied to all other images, but this can also be performed manually. Image registration for different stains from the same block is automatically performed during import (from the scanner to the PACS system), so the pathologist does not have to wait for the registration algorithm to be applied when opening the case. Annotations can be made on the images and can be viewed by multiple observers, and a list of annotations is available through keyboard shortcuts. Tools are provided, such as a ruler (useful for measuring the depth of invasion or the distance to the resection margin), arrows (for highlighting specific parts of the slide), and rectangular and polygonal shapes (useful for focusing the attention of viewers, e.g. during multidisciplinary meetings, or for documenting important diagnostic features) (Figure 8). Furthermore, a mitotic count tracker is included, which locks the screen at ×40 magnification. During scrolling through breast tissue and marking mitoses, the tool keeps track of the number of marked mitoses and the area covered, and gives a signal upon reaching 1.00 and 2.00 mm^2^. This provides an easy way to assess mitotic count, e.g. for Bloom–Richardson grading of breast carcinomas and sarcomas. Finally, a Ki67 quantification algorithm is available for assessing the percentage of Ki67‐positive cells in a selectable total number of nuclei.

**Figure 9:**
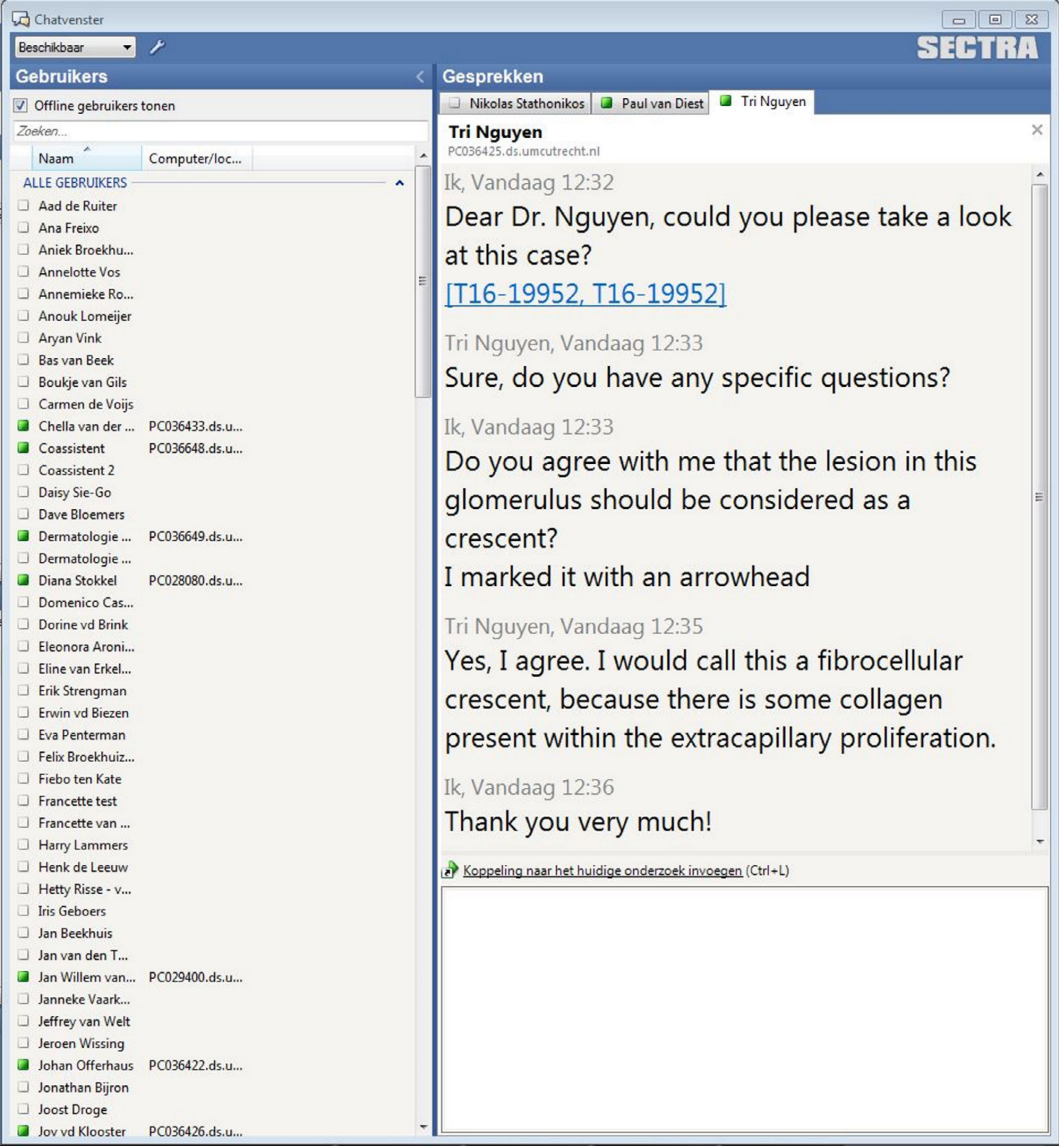
Example of a digital case chat.

Furthermore, the PACS system is equipped with a chat function, whereby digital links to cases can be provided, allowing for internal consultation between colleagues (Figure 9). Moreover, multiple pathologists can evaluate the same slide at once, while the ‘presence’ of other viewers is shown, allowing live discussions. The chat discussion can be performed interactively, as each others' arrows are visible while further chat comments are made.

The PACS system offers the possibility of constructing ‘dynamic worklists’, which are updated constantly on the basis of preselected criteria. We have made several of these worklists for organ specialists and priority cases. Apart from the general worklists, we have also made personalised worklists per specialist, which are updated as soon as a case is assigned to a specialist.

The PACS system is synchronised with our pathology reporting system (U‐DPS; PALGA, Houten, The Netherlands), so, as a case is selected in the PACS system, the same case is opened immediately in U‐DPS. All of the reporting is performed in U‐DPS, so, as soon as the report is authorised, the status of the case is synchronised in the PACS system, and the case is removed from the worklist. In this way, it is ensured that the worklist ‘empties’ as soon as a case is completed.

Initially, we expected that a digital workflow might result in a delay in diagnosing cases as compared with working with glass slides, because of the learning curve regarding working with a new system. Because of the high performance and user‐friendliness of the PACS system, the hardware choices made for the specialist workstations, and the choices made for server hardware, we managed to construct a high‐performance system, which resulted in the removal of diagnostic delays. Although no formal time registrations have been performed, the general feeling is that cases can be diagnosed more quickly with digital microscopy than with light microscopy.

### Laboratory Workflow

As soon as the slides are stained and coverslipped, they are placed on a special airflow‐based drying table to accelerate the drying of the mounting medium, which, when it is not completely dry, can result in a mechanical risk to the slide scanners. Also, we switched back from a xylene replacement to xylene to speed up drying. At this point, a quality assurance step and the slide case assembly are performed. Before scanning, the slides are carefully checked for sticky mounting medium remnants and cleaned. As soon as the case is complete, the slides are moved to the scanning room, where they are placed in a queue to be scanned. At present, the case assembly of the H&E‐stained slides is performed before they are transferred for scanning, impeding somewhat the continuous workflow of the process. IHC stained slides and special stainings are sent as separate batches as soon as they are ready, in a similar fashion. This is an artificial restriction on the workflow to assist the laboratory technicians in transitioning to a fully digital workflow for the first year of the implementation. After all of the minor details are worked out and the laboratory is comfortable with the new workflow, the slides will be moved immediately after staining to the scanning area and placed into a queue, and the case assembly will be performed digitally. The PACS system will be informed by the LIMS on how many images to expect and whether the case is complete or not. As soon as the case is complete, a final check is performed digitally by a technician. The images are compared with the slide to ensure that all tissue has been scanned, and the quality of the images is assessed. If deemed necessary, immediate rescans are performed with more or different focus points or another scanning protocol. The scanners also have an automated quality control/rescan option. Obviously bad images are removed by the scanning technicians, and rescans go into the PACS system for the pathologists and residents to choose from, and delete the worst ones. After a case has been completed, it will be released to the PACS system by the scanning technicians.

**Figure 10:**
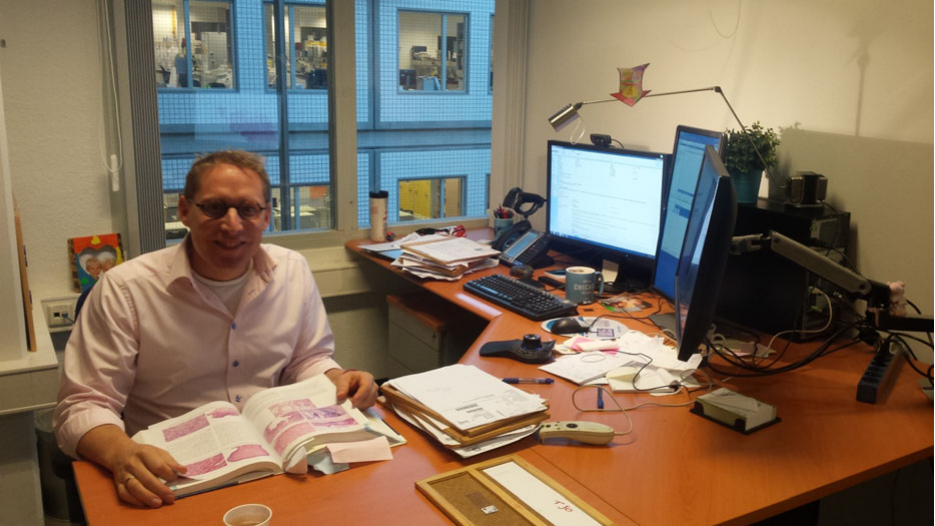
Dr Stefan Willems, the first microscope‐less pathologist at the University Medical Centre Utrecht.

For the first year of implementation, we opted for a double workflow, meaning that cases are booked out both digitally and with the traditional method—handing out the glass slides. This is a deliberate decision to support the transition and adaptation to the new workflow, and it is part of our extensive change management efforts focused on bringing this project to success. The motivation behind this step is to offer specialists the option to choose which method they prefer for diagnosing cases. Our experience so far ranges from an enthusiastic switch to a complete digital workflow for most, to using the PACS system together with a traditional workflow for reviewing patient history. So far, no pathologists have refused to use the PACS system, which we considered to be a possibility before starting the project. For techniques that are not supported by the whole slide scanner, e.g. polarisation, pathologists still use the microscope in their own room or in the diagnostics room. Levels of enthusiasm vary from reserved, to true enthusiasts who have even had their microscopes removed (Figure 10).

### Change Management

For an optimal change management process in which this project is formally introduced to all employees at our department, and to ensure that all stakeholders were closely involved, we have set up a project organisation consisting of a steering committee, a project group, and five different working groups. The working groups consisted of members of all subunits of our department, together with representatives from Sectra AB and Visiopharm, and were involved in quality, workflow, digital workspace, technique, and image analysis. All working groups held regular meetings in which specific tasks were effectuated into concrete action lists with main deliverables and deadlines. An important role of each working group was to inform colleagues with regular updates by providing presentations and interactive demonstrations to the department. Before the implementation of the project, all (end) users at the department were given training sessions in small groups.

This structure appeared to work very well. The subgroups were given clear but fairly abstract goals (e.g. ‘set up slide‐scanning logistics’), but, at the same time, the autonomy to work in their own way, and discover and solve problems on their own. This created true ownership of problems and the required commitment to be successful. Rarely, escalation to higher management levels was necessary.

### The Business Case

Motivated by studies that have demonstrated the reductions in cost, the increase in the quality of diagnosis and the reduction of errors that can be achieved by implementing a fully digital workflow,^17^ we decided to take the next step towards full digitisation. Unfortunately, however, our hospital does not provide an extra budget for technological innovations. We therefore concluded that a business case did not have much use. Instead, we decided to stick with our urge for innovation in pathology diagnostics (as before in the implementation of next‐generation sequencing^18^) and to follow our vision to create a breakthrough in digital pathology diagnostics. We therefore simply calculated what we could afford and wanted to spend over a period of 5 years with respect to our investment and consumables budgets, to set a maximum ‘total cost of ownership’, and assessed whether companies would be interested in this unique project on the basis of knowledge of list market prices for scanners and workflow software. This means that this whole project was realised with our own financial strength. In the future, we expect to obtain a realistic idea of the return on investment with regard to gains in efficacy and the quality of work. At this point, the whole digital operation, including storage, is estimated to cost <3% of our yearly budget. This may increase slightly because the number of slides to be scanned has risen since the beginning of the project, especially specials such as large 2 × 3 slides and fluorescence slides. Currently, we possess only one smaller scanner that scans these two types of slide; this has limited capacity, and a second one will be necessary soon.

## Methods

After 2 years of working with the system, we sent out a survey to all of our pathologists and residents to ask them their opinion on working with the digital system. The survey covered questions regarding three themes: usage of digital microscopy, i.e. how it is utilised within the context of their daily routine, ergonomics, and perceived quality for digital diagnostics.

We also calculated the turnaround time for routine diagnostic cases before (2015) and after (2016) implementation of the digital pathology workflow. We gathered the turnaround time per case for 2015 and 2016, and compared the mean turnaround time per weighted category as well as the total turnaround time. The cases were divided into six categories on the basis of a weighted index that indicates the severity of every case. The categories range from 1 to 6, where 1 indicates routine cases that need no extra tests, and 6 indicates the most complicated cases, which require multiple extra tests. The division of cases into categories is defined by a national protocol^19^ of the National Association of Pathologists in The Netherlands. We calculated the turnaround time by subtracting the date on which the report was authorised from the date on which the specimen was registered in our system. The turnaround time was measured in working days.

## Results

### Survey

We received survey input from 23 individuals with various degrees of experience in pathology and various amounts of exposure to digital pathology systems. All respondents had at least 6 months of experience working with the system. We opted to assess their confidence level in interacting with the system and requested feedback when they felt that the system was not yet optimal. The most important metric for this project was assessing the confidence level of the respondents concerning working with a digital microscopy system for diagnostic purposes.

The majority of the respondents felt either very confident (43.5%) or rather confident (30.4%) in working digitally, with only a few respondents feeling slightly confident (4.3%) or not confident at all (4.3%). The people who responded that the quality of scanned slides is good enough only for a minority of cases were mainly working in paediatrics, haematopathology, and neuropathology. Pathologists and residents from all other specialties responded that the quality of scanned slides was good enough for either the majority of or all primary diagnostic cases. The most frequent complaint that pathologists had regarding the quality of scanned slides was that it was difficult to discriminate microorganisms and mitoses. Dermatopathologists noted the low contrast for PAS‐D stains performed for the detection of fungus. These issues can probably be improved by Z‐stacking or scanning at higher resolution, at the cost of longer scanning times and more storage, or the implementation of machine‐learning algorithms, which we are currently pursuing. Neither the age of respondents nor the number of years of pathology experience was correlated with the confidence level regarding digital diagnostics.

Regarding ergonomics, most respondents found digital microscopy to be ergonomic (47.8%) to very ergonomic (17.4%), and only few found it to be fairly ergonomic (26.1%) to slightly ergonomic (8.7%). Respondents indicated fewer injuries related to digital microscopy than to traditional microscopy (Table 4).

**Table 4:** Frequencies of injury for traditional microscopy versus digital diagnostics as reported by 23 respondents

Injuries	Microscope, *n* (%)	Digital, *n* (%)
Head/neck	8 (35)	5 (22)
Shoulder	9 (39)	5 (22)
Wrist	3 (13)	4 (17)
Lower back	5 (22)	5 (22)
Legs/feet	1 (4)	1 (4)

### Turnaround Time

We calculated the turnaround time per severity category for all routine histological cases, excluding revisions and consultations (Table 5). Overall, the turnaround time decreased from 6.16 days to 5.73 days (6.94%), with the largest effect being seen in the complex cases categories. For the most complex category (6), the mean turnaround time decreased by 20.16%, which is almost 2 days less per case.

**Table 5:** Turnaround times divided per weight category (1 = simple case; 6 = most complex case) for 2015 (before) and 2016 (after) implementation of digital pathology

	2015		2016	
Weight category	No. of cases	Turnaround time (days)	No. of cases	Turnaround time (days)
1	8363	4.73	8470	4.78
2	9979	4.87	10 964	5.14
3	4427	8.00	4636	6.65
4	1593	10.71	1621	7.29
5	1652	9.63	1716	9.05
6	1066	9.66	1060	7.72

## Discussion

The introduction of fast and affordable whole slide scanners has facilitated the implementation of digital pathology with various use cases, the final challenge probably being full digital diagnostics. At the UMC Utrecht, The Netherlands, we took this final step in 2015 as one of the first laboratories to go fully digital with a future‐proof complete digital archive. The system was successfully implemented in ~6 months, including a European tender procedure. Most pathologists and residents had high confidence in working fully digitally, the expertise areas lagging behind being paediatrics, haematopathology, and neuropathology. Reported limitations concerned, in particular, recognition of microorganisms and mitoses.

After going live, we ran parallel glass slide and digital workflows for ~1 year to allow a smooth transition to working primarily digitally, and left it to the subspecialty teams to decide when to abandon the glass slide routine, which was achieved for most subspecialties. Interestingly, paediatric pathologists, haematopathologists and neuropathologists remained reluctant to work fully digitally; this was related to perceived limitations in image quality with regard to colour, microorganisms, mitoses, and nuclei in placental erythrocytes. Future improvements in scanner image quality will be needed to solve these problems, as preliminary testing of other current scanners has not shown spectacularly better results. As occasional cases or slides with suboptimal image quality (or the need for birefringence) occur across subspecialties, we have a service in place to quickly deliver the glass slides when ordered.

Interestingly, neither age nor the number of years of pathology experience was correlated with the confidence level regarding digital diagnostics. This may indicate that the ability to absorb change is a mindset rather than an age factor. The ergonomics of digital diagnostics were better than those with traditional microscopy, which is one of the secondary advantages of going digital, indirectly contributing to return on investment by, probably, reducing sick days. Our perception that digital diagnostics is, overall, faster than working with the microscope is in line with a recent study on time savings through digital pathology^20^ as well as our results regarding turnaround times when we compared the year before with the year after implementation of the system. Although it is hard to attribute the reduction in turnaround times solely to the introduction of the digital pathology system, we assume that it has significantly contributed to it. Apart from the direct effect (shorter access times, immediate access to slide data, and better sharing of the diagnostic load through worklists), there is an indirect effect resulting from reviewing the workflow. In order to implement the changes needed, we had to inventory all processes running in our laboratory, and we could quickly address inefficiencies regardless of their relationship with the implementation of the digital system.

In collaboration with Sectra, tweaking of the viewer and workflow system has been a continuous process. In the meantime, viewing tracking, storage of chat dialogues and storing observer credentials with the annotations have been realised, and further optimisations are anticipated. We aim at full bidirectional connections between the PACS system and U‐DPS and LIMS, which will further increase patient safety. Furthermore, the full switch to DICOM format directly from the scanners will need to be realised to arrive at a vendor‐independent future‐proof archive. The ‘intelligent lifecycle management’ solution, although technically available (e.g. strip the ×40 layer after a year), has not yet been implemented, because we hope to use a new much cheaper central storage solution at the UMC Utrecht in 2019, which may allow us to keep all files in the original ×40 resolution while still being affordable. After a few years of experience, we remain convinced that retaining a complete digital archive is an important part of the ‘return on investment’ of going digital. One of the planned improvements is full integration of the WSIs in the hospital information system, to the point that even patients can see their own images through the patient portal of the UMC Utrecht.

Furthermore, we are currently exploring ways of integrating in‐house‐developed^21^ deep‐learning algorithms and Visiopharm image‐processing algorithms for routine diagnostic work in order to determine how effective they would be in practice. Finally, we have started working on the implementation of Z‐scanning for cytology.^22^, ^23^ When Z‐stacks appear to be necessary, this will have consequences for our scanning and storage workflow.

The process of digitising a pathology laboratory for tasks other than primary diagnostics can probably be treated as a regular acquisition of equipment, and can usually be performed without disturbing regular workflow patterns. However, as soon as primary diagnostics becomes the main target of digitisation, a situation is created that affects every single aspect of the workflow. We show here that, with a good implementation plan and careful change management, a fully digital workflow can successfully be implemented in a relatively short period of time. After 2 years of experience, we believe that, in view of the many advantages, the changes needed for digital primary diagnostics are well worth the investment in capital (both human and monetary), which will place the laboratory in a situation to welcome promising technology in the near future while familiarising the staff with current technology. A laboratory that can keep a digital archive of all of its cases and perform diagnostics digitally is a laboratory that will benefit the most from technologies such as machine‐learning and image‐processing applications, and will reap all the benefits that accompany full digitisation.

## Conflict of interest

The authors state that they have no conflicts of interest.

## Author contributions

All authors contributed to the writing and editing of the article.

## References

[1] Huisman A , Looijen A , van den Brink SM *et al* Creation of a fully digital pathology slide archive by high‐volume tissue slide scanning. Hum. Pathol. 2010; 41; 751–757. 20129646 10.1016/j.humpath.2009.08.026

[2] Dackus GMHE , Jóźwiak K , Sonke GS *et al* Optimal adjuvant endocrine treatment of ER+/HER2+ breast cancer patients by age at diagnosis: a population‐based cohort study. Eur. J. Cancer 2018; 90; 92–101. 29274928 10.1016/j.ejca.2017.11.010

[3] Valkenburg M , Dijk M , ten Cate O *et al* Traditionele versus virtuele microscopie bij het onderwijs in de histopathologie. Tijdschr. voor Med. Onderwijs 2009; 28; 261–268.

[4] Hamilton PW , Wang Y , McCullough SJ . Virtual microscopy and digital pathology in training and education. APMIS 2012; 120; 305–315. 22429213 10.1111/j.1600-0463.2011.02869.x

[5] Vitkovski T , Bhuiya T , Esposito M . Utility of telepathology as a consultation tool between an off‐site surgical pathology suite and affiliated hospitals in the frozen section diagnosis of lung neoplasms. J. Pathol. Inform. 2015; 6; 55. 26605120 10.4103/2153-3539.168515 PMC4639948

[6] Holten‐Rossing H , Larsen LG , Toft BG *et al* Consultation on urological specimens from referred cancer patients using real‐time digital microscopy: optimizing the workflow. J. Pathol. Inform. 2016; 7; 11. 27076989 10.4103/2153-3539.177689 PMC4809123

[7] Al‐Janabi S , Huisman A , Vink A *et al* Whole slide images for primary diagnostics of gastrointestinal tract pathology: a feasibility study. Hum. Pathol. 2012; 43; 702–707. 21937077 10.1016/j.humpath.2011.06.017

[8] Al‐Janabi S , Huisman A , Nikkels PGJ *et al* Whole slide images for primary diagnostics of paediatric pathology specimens: a feasibility study. J. Clin. Pathol. 2013; 66; 218–223. 23204560 10.1136/jclinpath-2012-201104

[9] Al‐Janabi S , Huisman A , Vink A *et al* Whole slide images for primary diagnostics in dermatopathology: a feasibility study. J. Clin. Pathol. 2012; 65; 152–158. 22031590 10.1136/jclinpath-2011-200277

[10] Al‐Janabi S , Huisman A , Willems SM *et al* Digital slide images for primary diagnostics in breast pathology: a feasibility study. Hum. Pathol. 2012; 43; 2318–2325. 22901465 10.1016/j.humpath.2012.03.027

[11] Dackus GM , Ter Hoeve ND , Opdam M *et al* Long‐term prognosis of young breast cancer patients (≤40 years) who did not receive adjuvant systemic treatment: protocol for the PARADIGM initiative cohort study. BMJ Open 2017; 7, e017842. 10.1136/bmjopen-2017-017842 PMC5695414 29138205

[12] Al‐Janabi S , Huisman A , Van Diest PJ . Digital pathology: current status and future perspectives. Histopathology 2012; 61; 1–9. 10.1111/j.1365-2559.2011.03814.x 21477260

[13] Williams BJ , DaCosta P , Goacher E *et al* A systematic analysis of discordant diagnoses in digital pathology compared with light microscopy. Arch. Pathol. Lab. Med. 2017; 141; 1712–1718. 28467215 10.5858/arpa.2016-0494-OA

[14] Stathonikos N , Veta M , Huisman A *et al* Going fully digital: perspective of a Dutch academic pathology lab. J. Pathol. Inform. 2013; 4; 15. 23858390 10.4103/2153-3539.114206 PMC3709427

[15] Storteboom A , Wondimu P , Lohne J *et al* Best value procurement – the practical approach in the Netherlands. Procedia Comput. Sci. 2017; 121; 398–406.

[16] Randell R , Ambepitiya T , Mello‐Thoms C *et al* Effect of display resolution on time to diagnosis with virtual pathology slides in a systematic search task. J. Digit. Imaging 2014; 28; 68–76. 10.1007/s10278-014-9726-8 PMC4305057 25128321

[17] Ho J , Kuzmishin J , Montalto M *et al* Can digital pathology result in cost savings? A financial projection for digital pathology implementation at a large integrated health care organization. J. Pathol. Inform. 2014; 5; 33. 25250191 10.4103/2153-3539.139714 PMC4168664

[18] De Leng WWJ , Gadellaa‐Van Hooijdonk CG , Barendregt‐Smouter FAS *et al* Targeted next generation sequencing as a reliable diagnostic assay for the detection of somatic mutations in tumours using minimal DNA amounts from formalin fixed paraffin embedded material. PLoS One 2016; 11; e0149405. 26919633 10.1371/journal.pone.0149405 PMC4769293

[19] Zwaarte categorieën ‐ pathology.nl. [Accessed July 2, 2019] Available at: https://pathology.nl/vereniging/commissies/beroepsbelangen/zwaarte-categorieen/

[20] Baidoshvili A , Bucur A , van Leeuwen J *et al* Evaluating the benefits of digital pathology implementation: time savings in laboratory logistics. Histopathology 2018; 73; 784–794. 29924891 10.1111/his.13691

[21] Veta M , van Diest PJ , Willems SM *et al* Assessment of algorithms for mitosis detection in breast cancer histopathology images. Med. Image Anal. 2015; 20; 237–248. 25547073 10.1016/j.media.2014.11.010

[22] Bongaerts O , Clevers C , Debets M *et al* Conventional microscopical versus digital whole‐slide imaging‐based diagnosis of thin‐layer cervical specimens: a validation study. J. Pathol. Inform. 2018; 9; 29. 30197818 10.4103/jpi.jpi_28_18 PMC6120269

[23] Bongaerts O , van Diest P , Pieters M *et al* Working toward consensus among professionals in the identification of classical cervical cytomorphological characteristics in whole slide images. J. Pathol. Inform. 2015; 6; 52. 26605117 10.4103/2153-3539.166013 PMC4629309

